# The effect of 10 Hz transcranial alternating current stimulation (tACS) on corticomuscular coherence

**DOI:** 10.3389/fnhum.2013.00511

**Published:** 2013-08-29

**Authors:** Claudia Wach, Vanessa Krause, Vera Moliadze, Walter Paulus, Alfons Schnitzler, Bettina Pollok

**Affiliations:** ^1^Institute of Clinical Neuroscience and Medical Psychology, Medical Faculty, Heinrich-Heine-UniversityDuesseldorf, Germany; ^2^Department of Neurology, Medical Faculty, Duesseldorf University HospitalDuesseldorf, Germany; ^3^Department of Child and Adolescent Psychiatry, Psychosomatics and Psychotherapy, Goethe-University of Frankfurt am MainFrankfurt am Main, Germany; ^4^Department of Clinical Neurophysiology, Georg-August-UniversityGoettingen, Germany

**Keywords:** transcranial alternating current stimulation (tACS), magnetoencephalography (MEG), corticomuscular coherence (CMC), motor control, primary motor cortex (M1)

## Abstract

Synchronous oscillatory activity at alpha (8–12 Hz), beta (13–30 Hz), and gamma (30–90 Hz) frequencies is assumed to play a key role for motor control. Corticomuscular coherence (CMC) represents an established measure of the pyramidal system's integrity. Transcranial alternating current stimulation (tACS) offers the possibility to modulate ongoing oscillatory activity. Behaviorally, 20 Hz tACS in healthy subjects has been shown to result in movement slowing. However, the neurophysiological changes underlying these effects are not entirely understood yet. The present study aimed at ascertaining the effects of tACS at 10 and 20 Hz in healthy subjects on CMC and local power of the primary sensorimotor cortex. Neuromagnetic activity was recorded during isometric contraction before and at two time points (2–10 min and 30–38 min) after tACS of the left primary motor cortex (M1), using a 306 channel whole head magnetoencephalography (MEG) system. Additionally, electromyography (EMG) of the right extensor digitorum communis (EDC) muscle was measured. TACS was applied at 10 and 20 Hz, respectively, for 10 min at 1 mA. Sham stimulation served as control condition. The data suggest that 10 Hz tACS significantly reduced low gamma band CMC during isometric contraction. This implies that tACS does not necessarily cause effects at stimulation frequency. Rather, the findings suggest cross-frequency interplay between alpha and low gamma band activity modulating functional interaction between motor cortex and muscle.

## Introduction

Synchronized oscillatory activity subserves functional communication within and between brain sites (Schnitzler and Gross, 2005; Thut and Miniussi, 2009). During rest spontaneous oscillatory activity at alpha (8–12 Hz) and beta (13–30 Hz) frequency is prominent in the sensorimotor cortex (Salmelin and Hari, 1994) and has originally been considered as a correlate of inactive cortical areas (Salmelin et al., 1995; Sauseng et al., 2009). Recent data suggest that information gating is mediated by inhibition of task-irrelevant brain regions reflected by oscillatory activity in the alpha band, whereas active processing appears to be associated with gamma band synchronization at frequencies between 30 and 100 Hz (Jensen and Mazaheri, 2010).

Dynamic functional interaction in a cerebello-thalamo-cortical network underlying motor control has been evidenced at alpha and beta frequencies (Gross et al., 2002; Pollok et al., 2004, 2005). Gamma oscillations in the primary motor cortex (M1) have been observed particularly at movement onset (Crone et al., 1998; Pfurtscheller et al., 2003; Miller et al., 2007; Muthukumaraswamy, 2010) and are assumed to encode information regarding limb movement rather than muscle activity (Muthukumaraswamy, 2010). Corticomuscular coherence (CMC) represents an established neurophysiological marker of the pyramidal system's integrity reflecting functional coupling between M1 and muscles. It is particularly evident during isometric contraction mainly at the beta range (Conway et al., 1995; Salenius et al., 1997; Halliday et al., 1998; Brown, 2000; Kilner et al., 2000; Kristeva-Feige et al., 2002; Riddle and Baker, 2006; Baker, 2007; Kristeva et al., 2007; Witte et al., 2007), but at gamma frequency as well (Salenius et al., 1997; Brown, 2000; Gross et al., 2005). While beta band CMC is associated with static movements, gamma band CMC has been related to the control of dynamic movements (Brown and Marsden, 1998; Marsden et al., 2000; Schoffelen et al., 2005; Omlor et al., 2007; Chakarov et al., 2009). High gamma band CMC (60–100 Hz) was found during the execution of phasic movements (Marsden et al., 2000), whereas an increase of low gamma band CMC (40–70 Hz) accompanied selective movement preparation in a reaction-time task (Schoffelen et al., 2011). Additionally, gamma band CMC at 30–45 Hz was shown during isometric contraction requiring compensation of a periodically modulating force (Andrykiewicz et al., 2007; Omlor et al., 2007; Patino et al., 2008). The authors assumed an association of low gamma band CMC with information integration essential to generating the appropriate force adaptation.

The functional importance of alpha, beta, and gamma oscillations is underlined by findings regarding pathological changes of oscillatory activity, e.g., in Parkinson's disease (PD) (Brown, 2003; Schnitzler et al., 2006; Weinberger et al., 2009). CMC during isometric contraction is reduced in the beta and gamma band OFF and remedied ON medication (Salenius et al., 2002). Akinesia and rigidity as clinical hallmarks of PD have been related to exaggerated beta band oscillations particularly within the subthalamic nucleus (STN) (Jenkinson and Brown, 2011). Besides, experimentally enhanced 10 Hz oscillations within the STN by means of deep brain stimulation (DBS) yield worsening of PD symptoms (Timmermann et al., 2004).

Transcranial alternating current stimulation (tACS) provides the unique possibility of non-invasively modulating ongoing oscillatory activity in a frequency-specific way (Zaehle et al., 2010; Polanía et al., 2012) which has been attributed to oscillatory entrainment by the specific stimulation frequency (Zaehle et al., 2010). On the behavioral level, differential effects of stimulation frequency on motor functions have been shown (Wach et al., 2013): While 10 Hz tACS increased movement variability, 20 Hz tACS led to movement slowing. Accordingly, Pogosyan et al. (2009) showed an increase of beta band CMC during 20 Hz tACS accompanied by slowing of dynamic movements in healthy subjects. A recent study suggests that behavioral effects exceed the stimulation period and may even occur with a temporal delay of 30 min after tACS cessation (Wach et al., 2013). Interestingly enough, these results are less likely to be explained by entrainment and imply that tACS may induce plastic alterations yielding offline effects of stimulation.

Several tACS studies converge toward entrainment effects to occur during tACS possibly even outlasting the stimulation for about 30 min (Herrmann et al., 2013). However, with prolonged brain stimulation there is an increased likelihood of observing prolonged offline effects brought about by other mechanisms such as changes in synaptic plasticity which may be orthogonal to the online entrainment effects. For instance, transcranial magnetic stimulation (TMS) has been suggested to lead to both entrainment and synaptic changes, but at different time scales, i.e., online vs. offline (Thut and Pascual-Leone, 2010; Thut et al., 2011). Yet, the exact neurophysiological mechanisms underlying online and offline effects of non-invasive brain stimulation remain to be solved.

Against this background the present study aims at ascertaining the effects of 10 and 20 Hz tACS on local M1 oscillatory activity and CMC during isometric contraction in healthy subjects in order to elucidate the functional relevance of these frequencies for motor control. Furthermore, the study intends to shed further light on neurophysiological mechanisms underlying offline effects of tACS such as oscillatory entrainment or neuroplastic alterations. As offline effects may be indicative of neural plasticity and particularly important in view of potential therapeutic applications of tACS, we focused on the investigation of such offline effects using magnetoencephalography (MEG). This method allows the non-invasive measurement of neuromagnetic brain activity with an excellent temporal resolution (Hämäläinen et al., 1993; Hari, 2005).

## Materials and methods

### Ethics statement

The study was in accordance with the latest revision of the *Declaration of Helsinki*. Experimental procedures were approved by the local ethics committee of the Heinrich-Heine-University, Duesseldorf, Germany. Prior to the experiment subjects gave their written informed consent.

### Subjects

Twenty healthy subjects (8 males) aged between 19 and 60 (28.6 ± 2.0; mean ± standard error of mean; SEM) years were screened for showing a distinctive beta band CMC during isometric contraction. This procedure was chosen because not all subjects display a prominent CMC (Mima et al., 2000; Baker and Baker, 2003; Hashimoto et al., 2010; Mendez-Balbuena et al., 2012). To this end, CMC was assessed via visual inspection using the MEG analysis software Graph (Elekta Neuromag Oy, Helsinki, Finland).

Twelve subjects (5 males) aged between 22 and 60 (30.1 ± 3.0) years were eligible for study participation. All participants were right-handed according to the Edinburgh Handedness Inventory (Oldfield, 1971). All subjects completed the entire study protocol. Exclusion criteria were history or family history of epileptic seizures, history of migraine, unexplained loss of consciousness, or brain related injury, history of other neurological or psychiatric disorders, intake of central nervous system-effective medication, cardiac or brain pacemaker, or metal implants (clips, cochlear implants, piercings, dental braces) that could not be removed for the duration of the experimental sessions.

### Transcranial alternating current stimulation

After localizing the forearm representation of left M1 by single TMS pulses, tACS was delivered by a DC stimulator (NeuroConn GmbH, Ilmenau, Germany) through a pair of saline-soaked rectangular sponge electrodes (5 × 7 cm). In accordance with a standard montage previously used (Nitsche and Paulus, 2000; Moliadze et al., 2010; Wach et al., 2013), one electrode was placed above left M1, whereas the other one was fixed over the contralateral orbit. Electrodes were held in place by rubber bands. Stimulation was applied at 10 and 20 Hz for 10 min, respectively. Peak-to-peak current intensity was 1 mA. Current was ramped up and down over the first and last 5 s of stimulation. Scalp current density below the stimulation electrode was 28.6 μA/cm^2^. Impedance was kept below 5 kOhm. Stimulation was in compliance with established safety protocols regarding DC and AC stimulation (Nitsche et al., 2003; Iyer et al., 2005). For sham stimulation tACS was applied for 30 s, only. Thus, subjects experienced the typical tingling sensation under the electrodes at the beginning of stimulation. Subjects and investigator were blind to tACS frequency.

In order to mask retinal stimulation effects associated with tACS frequencies below 45 Hz and an electrode montage above the orbit (Paulus, 2010), a visual 10 Hz flicker stimulus was displayed by a projector during each stimulation session (verum and sham). After each experimental session subjects completed a questionnaire regarding potential adverse effects such as itching, pain or skin irritation during and/or after stimulation. Moreover, they indicated whether they believed to have received verum or sham stimulation and evaluated the confidence of their decision by means of a numerical rating scale ranging from 1 (totally uncertain) to 10 (totally certain). If verum stimulation was chosen, subjects were asked to estimate stimulation frequency (10 vs. 20 Hz) and rate their estimation confidence again.

### Magnetoencephalography

Neuromagnetic activity was measured with a 306 channel whole head MEG system (Elekta Neuromag Oy, Helsinki, Finland), consisting of 204 planar gradiometers and 102 magnetometers. At the same time, surface electromyography (EMG) of the right extensor forearm muscle (extensor digitorum communis, EDC) using bipolar surface electrodes was recorded. In order to control for eye blink artifacts, vertical electrooculogram (EOG) was recorded. MEG and EMG data were measured with a sampling frequency of 1000 Hz, filtered with a band-pass filter of 0.03–330 Hz, and stored digitally for offline analysis. For the estimation of the head position with respect to the MEG sensor array magnetic signals from four indicator coils placed on the scalp were measured prior to each measurement. Coil localizations were identified with respect to nasion, left and right preauricular points serving as anatomical landmarks using a three-dimensional digitizer (Polhemus, Colchester, VT).

Neuromagnetic activity was recorded during isometric contraction of the right forearm and during rest. Isometric contraction was performed by lifting the right forearm at an angle of about 30 deg, keeping the hand outstretched and slightly abducting the fingers. The elbow remained on the armrest. Contraction was maintained at about 30% of maximum voluntary contraction for the duration of 1 min, while EMG activity was monitored online. Subsequently, the arm was put down and a 1 min period of rest followed. Isometric contraction and rest alternated four times amounting to a task duration of 8 min.

### Experimental design and procedure

A double-blind, randomized, sham-controlled within-subject design was implemented. Order of the three stimulation conditions (10 Hz, 20 Hz, sham) was counterbalanced across subjects. In half of the sample sham stimulation was applied at 10 and 20 Hz, respectively. Neuromagnetic activity during isometric contraction and rest of the right forearm was recorded before and at two time points after tACS (post1: 2–10 min and post2: 30–38 min). In order to avoid carryover effects, an interval of at least 1 week lay between the three experimental sessions. Subjects were naïve with respect to the exact aim of the study.

During MEG measurements the subjects were comfortably seated in a magnetically shielded room (MSR) keeping their eyes open during the measurement. Subsequently, tACS was applied outside the MSR. Afterwards subjects returned to the MSR and neuromagnetic activity was recorded again. The last measurement started 30 min after stimulation cessation.

### Data analysis

Analysis of MEG and EMG data was conducted using the Matlab (The Mathworks, Natick, Massachusetts, USA) based FieldTrip toolbox (Oostenveld et al., 2011). Only data from gradiometers were considered for the analysis. Data were segmented into time periods of isometric contraction and rest (4 × 1 min, respectively) according to the EMG signal. Movement periods were discarded by excluding the first and last three seconds of each segment from the analysis. EMG data were first high pass filtered at 60 Hz and then rectified. The filter was chosen instead of a baseline to correct for movement artifacts as a prerequisite for rectification. Alpha (8–12 Hz), beta (13–30 Hz), low gamma (30–45 Hz), and high gamma (45–90 Hz) bands were defined as frequencies of interest. Data were transformed from time into frequency domain by fast Fourier transformation (FFT). FFT size was 1000 samples. Cross-spectral density was calculated with a frequency resolution of 1 Hz, after applying a Hanning window for the analysis of oscillatory patterns below 30 Hz and a discrete prolate spheroidal sequence (dpss) taper for gamma frequencies. Smoothing of 2 Hz was performed for the analysis of oscillatory activity above 30 Hz.

**Figure 1:**
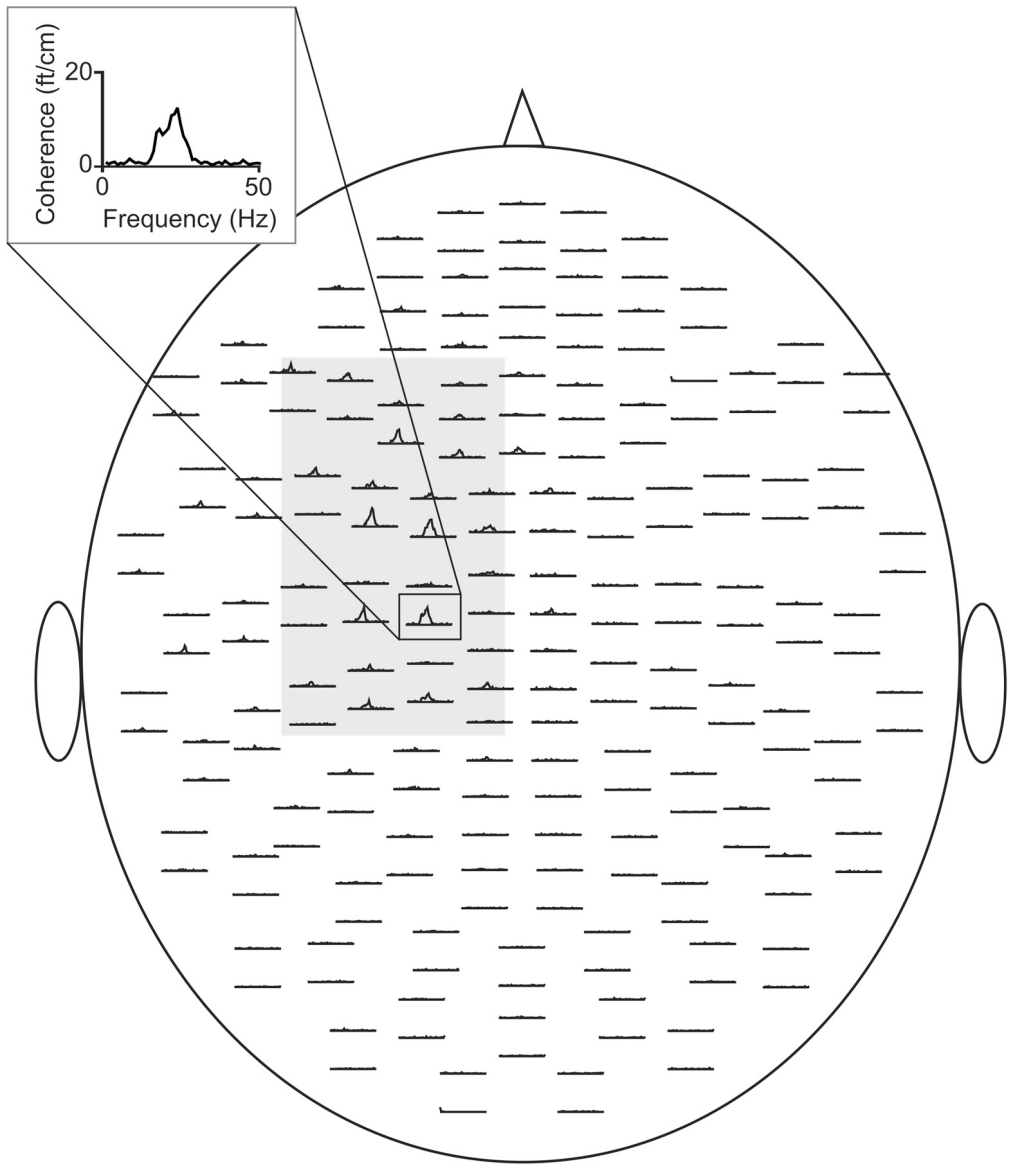
**CMC sensor-plot averaged across all subjects prior to tACS**. The insert indicates the sensor with the largest amplitude. The gray shaded area represents the sensors selected for the analysis. Data were averaged across this selection.

Analysis encompassed local power and coherence between MEG and EMG (i.e., CMC). Spectral power reflects synchronized oscillatory activity within local brain areas, whereas CMC represents an established measure of functional connectivity between M1 and contralateral muscles. It quantifies the functional interaction in the frequency domain and ranges from “0” (complete linear independence of signals) to “1” (perfect linear correlation). At each frequency of interest maximum peaks of spectral power and CMC were determined in individual data sets. Data were analyzed on the sensor level. To this end, channels covering the left sensorimotor cortex (Figure 1) were selected and data were averaged across this selection. Then, in each individual the main peak in each frequency band of interest was selected and the corresponding frequency and amplitude were determined.

### Statistics

All statistical comparisons were calculated using IBM SPSS Statistics 20. Data were tested for Gaussian distribution by means of Kolmogorov-Smirnov test. Outlier correction was performed by excluding values outside the 95% confidence interval (mean ± two standard deviations) and replacing them by column means. To account for pre-stimulus fluctuations and inter-individual variability, post tACS amplitude values were normalized to pre tACS by calculating relative changes with respect to individual pre tACS values for each of two post stimulation measurements.

Concerning CMC repeated analyses of variance (ANOVA) with factors *stimulation* (10 vs. 20 Hz vs. sham) and *time* (post1 (i.e., 2–10 min after tACS cessation) vs. post2 (i.e., 30–38 min after tACS cessation) were calculated. With regard to the analysis of power the factor *hemisphere* (ipsilateral vs. contralateral) was included additionally in order to account for potential laterality effects. Data were analyzed separately for each frequency band. Paired *t*-tests were employed for *post-hoc* analyses. In case of sphericity violation Greenhouse-Geisser-corrected *p*-values are given. *P*-values were adjusted for multiple testing.

## Results

### Stimulation questionnaire

TACS and TMS were tolerated well by all subjects. Eleven participants indicated a slight tingling sensation at the beginning of stimulation. One subject reported mild headache about 24 h after tACS. There were no further stimulation side-effects. Evaluation of the blinding procedure showed that subjects correctly identified sham stimulation in 42% of sessions with a mean subjective confidence rating of 5.5 ± 1.5. In 42% of verum sessions 10 Hz stimulation frequency was accurately classified with a mean confidence rating of 4.9 ± 1.0. Twenty hertz stimulation was correctly assessed in 50% with a mean confidence of 4.7 ± 0.6. All in all, correct assessment of stimulation condition corresponded to chance level and overall confidence rating was low suggesting that blinding was successful.

### MEG data

The investigation of tACS offline effects on CMC was supposed to be restricted to frequency bands significantly modulated by isometric contraction. In a first step, we tried to determine frequency bands with functional relevance for isometric contraction. We therefore calculated mean contralateral absolute CMC amplitude at alpha, beta, low and high gamma band during isometric contraction of the right forearm *prior to tACS* and during rest. Peak frequencies of power and CMC are illustrated in Table 1.

**Table 1:** **Peak frequencies (in Hz; mean ± SEM) of CMC and power in each frequency band during isometric contraction (pre vs. post1 vs. post2)**.

	**Alpha band**			**Beta band**			**Low gamma band**			**High gamma band**		
**tACS**	**Pre**	**Post1**	**Post2**	**Pre**	**Post1**	**Post2**	**Pre**	**Post1**	**Post2**	**Pre**	**Post1**	**Post2**
**CMC**												
10 Hz	10.3 ± 0.4	10.4 ± 0.4	10.3 ± 0.3	22.1 ± 0.9	22.0 ± 0.9	21.3 ± 1.2	33.8 ± 0.9	36.9 ± 1.2	35.3 ± 1.0	70.0 ± 3.2	65.0 ± 2.0	75.1 ± 3.0
20 Hz	10.7 ± 0.4	10.3 ± 0.5	10.1 ± 0.5	21.6 ± 1.0	21.8 ± 1.1	22.8 ± 0.9	35.5 ± 1.3	33.4 ± 0.9	34.0 ± 1.2	65.8 ± 2.1	67.5 ± 3.2	70.2 ± 3.2
sham	10.7 ± 0.3	10.5 ± 0.3	9.7 ± 0.5	22.0 ± 1.0	22.6 ± 0.9	21.3 ± 1.0	35.7 ± 1.5	36.3 ± 1.3	36.2 ± 1.4	72.4 ± 3.3	69.5 ± 3.5	73.8 ± 3.9
**POWER**												
10 Hz	9.3 ± 0.3	9.4 ± 0.3	9.5 ± 0.3	18.5 ± 0.6	18.5 ± 0.4	18.2 ± 0.5	42.8 ± 0.9	42.8 ± 0.9	43.0 ± 0.9	62.0 ±1.4	59.8 ± 1.3	61.2 ± 1.5
20 Hz	9.7 ± 0.3	9.7 ± 0.2	9.6 ± 0.3	18.3 ± 0.5	18.9 ± 0.6	17.8 ± 0.5	42.9 ± 1.0	44.0 ± 0.2	43.7 ± 0.1	58.4 ± 0.7	60.5 ± 1.3	59.9 ± 1.0
sham	9.4 ± 0.3	9.3 ± 0.3	9.4 ± 0.3	19.0 ± 0.6	18.4 ± 0.6	18.0 ± 0.6	43.5 ± 0.5	43.3 ± 0.7	43.0 ± 0.9	57.9 ± 0.9	61.2 ± 1.0	61.1 ± 1.2

**Figure 2:**
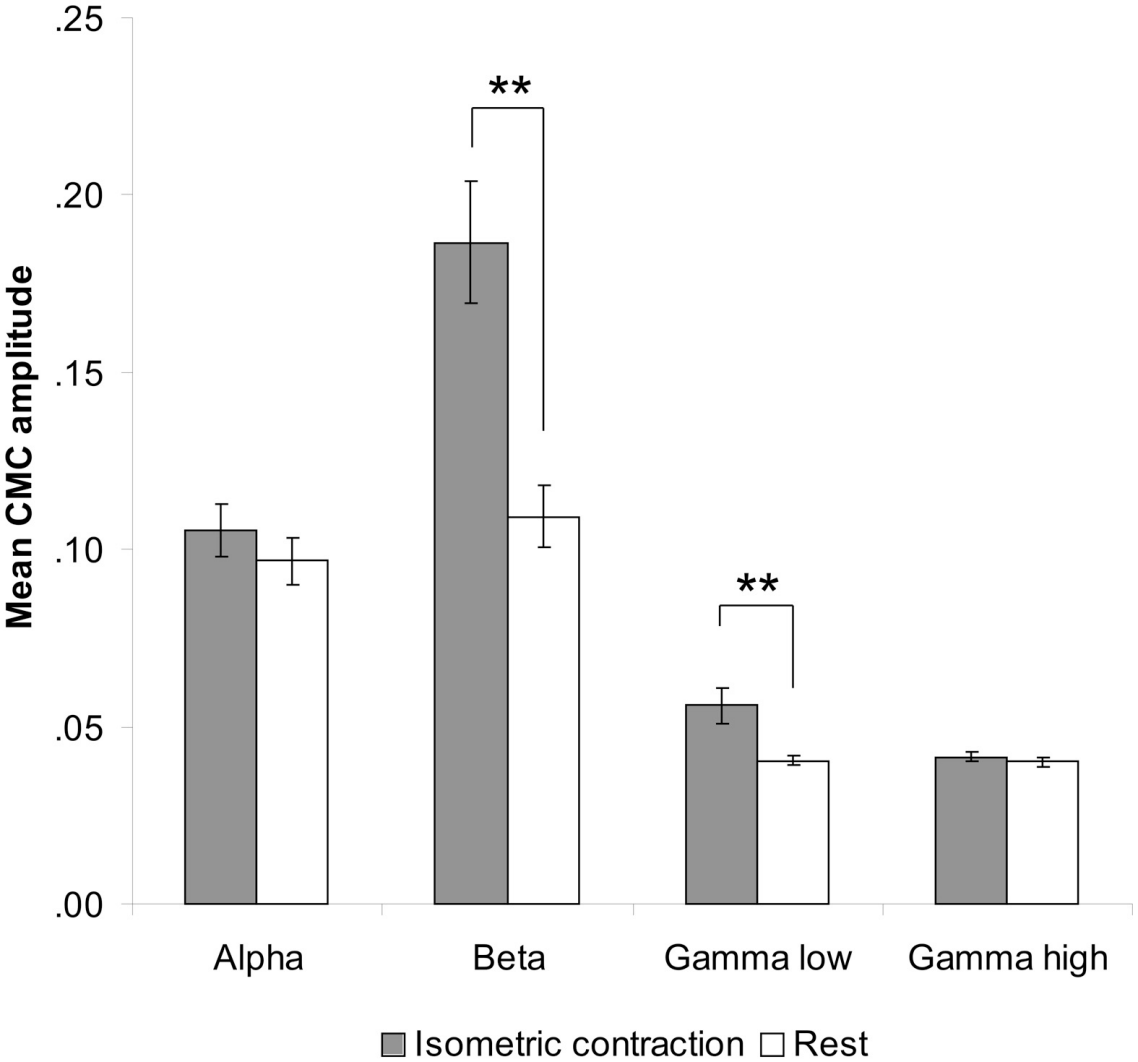
**Contralateral CMC amplitude during isometric contraction and rest prior to tACS**. A significant increase of CMC amplitude during isometric contraction compared to rest was only found in the beta (13–30 Hz) and low gamma (30–45 Hz) band. Error bars indicate standard error of mean. Asterisks denote significant *p*-values < 0.001.

Comparison between rest and isometric contraction revealed significant differences in the beta and low gamma band indicating stronger CMC during isometric contraction than during rest [beta band: *t*_(35)_ = 7.47, *p* < 0.001; low gamma band: *t*_(35)_ = 5.00, *p* < 0.001; Figure 2]. CMC at alpha and high gamma frequencies did not significantly differ between conditions, although a trend was observed (*p* > 0.090). We therefore restricted the subsequent CMC analysis to the beta and low gamma band. Since we cannot rule out the possibility that possible power modulations yielded by tACS at alpha and beta frequency might have effects on any of the selected frequency ranges, power analysis was extended to all four frequency bands (i.e., alpha, beta, low and high gamma).

CMC and power were analyzed with respect to the peak frequency in the respective frequency ranges of interest (i.e., the frequency with the largest amplitude) and the amplitude at this frequency. In the following we will use the term *frequency* when reporting the peak frequency and *amplitude* whenever we refer to the amplitude at this frequency.

### CMC

Regarding CMC frequency during isometric contraction no significant main effect of factor *stimulation* but a trend toward a significant s*timulation* × *time* interaction was evident at the beta range (*p* > 0.099). In the low gamma band a trend toward a significant s*timulation* × *time* interaction was revealed [*F*_(4, 44)_ = 2.41, *p* = 0.063]. However, *post-hoc* no significant effects were observed (*p* > 0.285).

**Figure 3:**
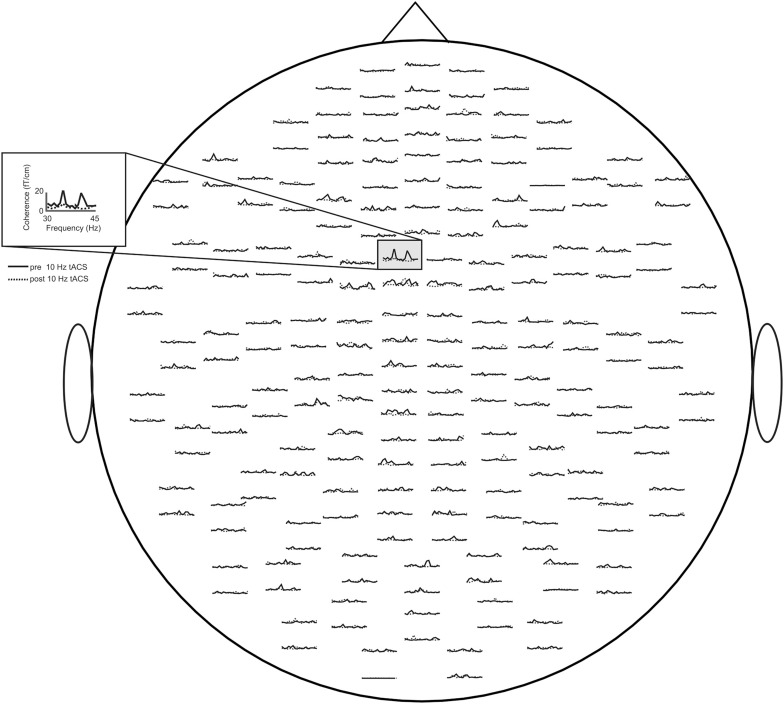
**Averaged sensor-plot for CMC within the low gamma band (30–45 Hz) prior to and post 10 Hz tACS**. The insert indicates the sensor with the largest amplitude change following tACS.

**Figure 4:**
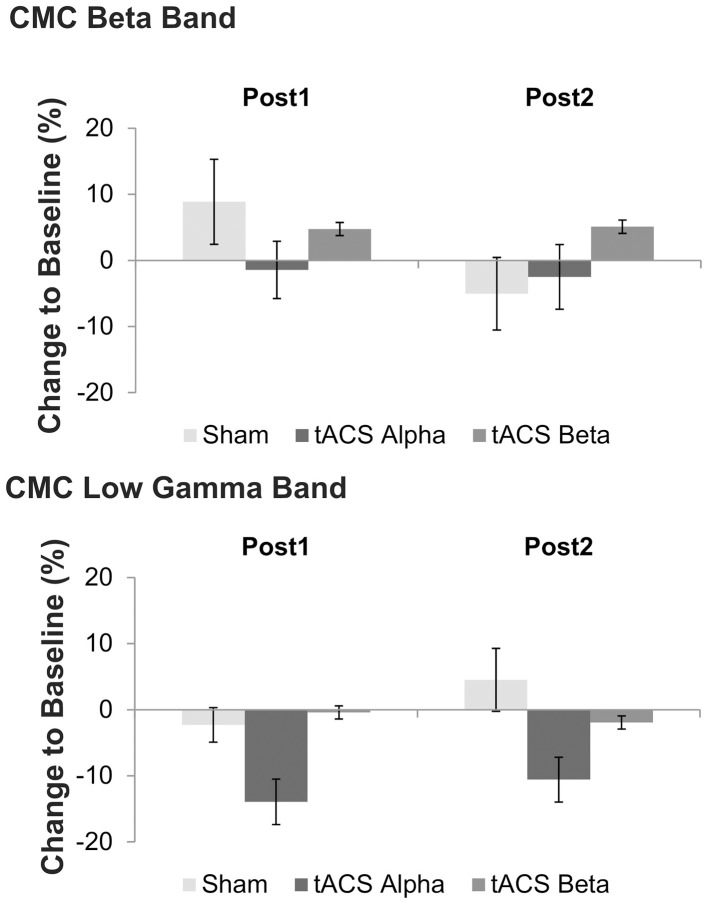
**Effects of tACS at 10 Hz, 20 Hz and sham stimulation on CMC at low gamma frequency at two time points after tACS cessation**. Shown are relative changes. Error bars indicate standard error of mean. Please note that a significant main effect of factor *stimulation* was found on low gamma band CMC, only suggesting more pronounced effects of 10 Hz tACS on low gamma CMC as compared to sham and 20 Hz stimulation.

**Figure 5:**
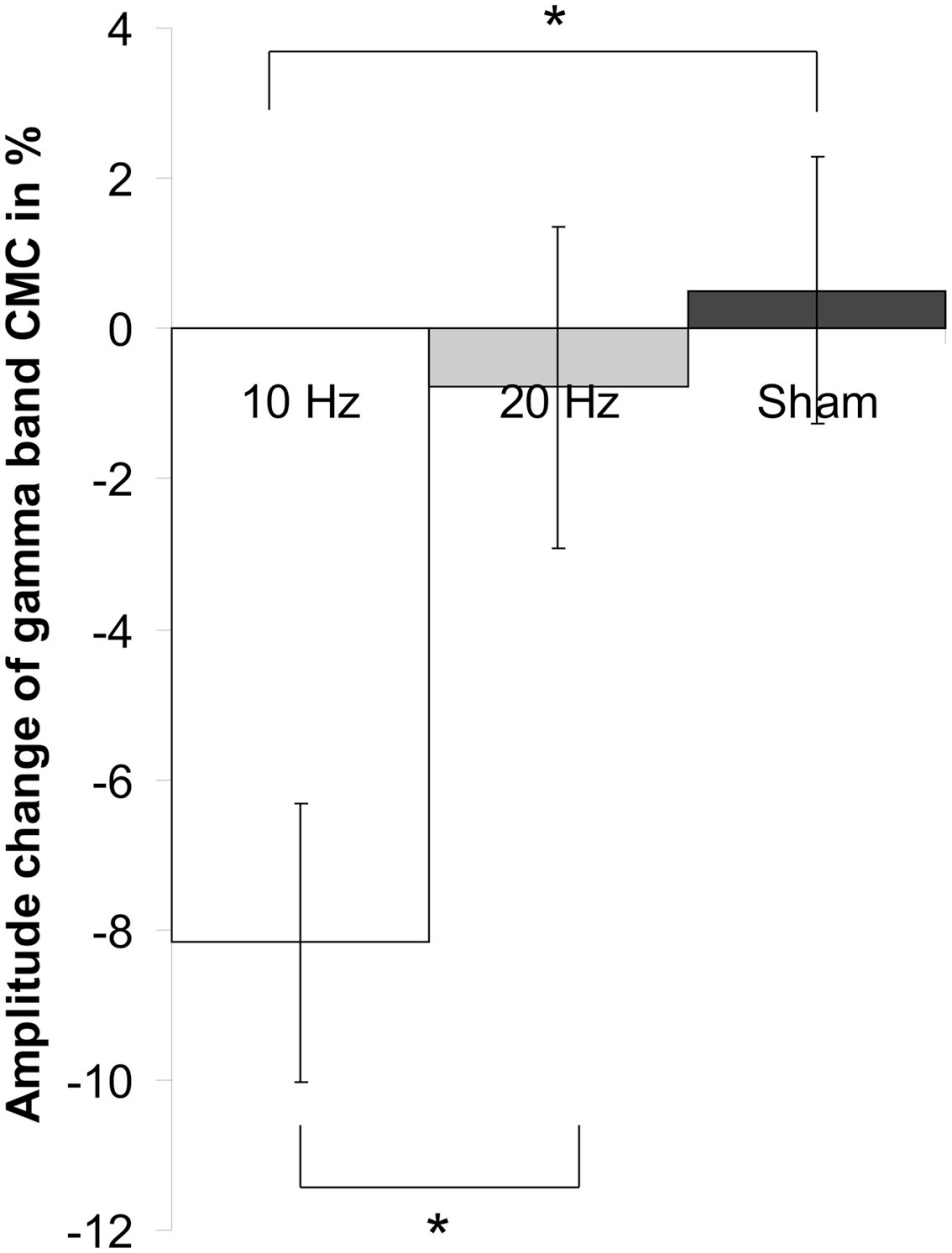
**Main effect of factor *stimulation* on low gamma CMC**. Shown are relative changes of contralateral CMC amplitude in the low gamma band during isometric contraction after 10 Hz, 20 Hz and sham tACS collapsed across the two post tACS measurements. After 10 Hz tACS CMC amplitude was significantly lower as compared to 20 Hz and sham stimulation. Asterisk denotes significant *p*-values < 0.05. Error bars indicate standard error of mean.

Concerning relative changes of CMC amplitude there was no significant main effect or interaction in the beta band (*p* > 0.11). In the low gamma band (Figure 3 depicts the sensor-plot) a significant main effect of factor *stimulation* was revealed [*F*_(2, 22)_ = 3.72, *p* = 0.041; Figure 4] suggesting that after 10 Hz tACS contralateral CMC amplitude was significantly reduced as compared to 20 Hz tACS and sham [10 Hz tACS vs. sham: *t*_(35)_ = −3.09, *p* = 0.008; 10 Hz tACS vs. 20 Hz tACS: *t*_(35)_ = −2.85, *p* = 0.021]. The main effect of factor *stimulation* is summarized in Figure 5. Neither a main effect of factor *time* [*F*_(1, 11)_ = 0.72, *p* = 0.41] nor the *stimulation x time* interaction [*F*_(2, 22)_ = 0.52, *p* = 0.60] was found to be significant.

### Power

We first compared absolute power values during isometric contraction and rest prior to tACS. The analysis revealed a significant decrease during isometric contraction at alpha [*t*_(34)_ = 3.28, *p* < 0.001] and beta [*t*_(34)_ = 2.99, *p* < 0.001] frequency, but neither at low [*t*_(34)_ = −0.56, *p* = 0.58] nor at high gamma frequencies [*t*_(34)_ = −1.10, *p* = 0.28].

During isometric contraction the analysis of relative power amplitude did neither reveal a significant main effect of factors *time* (*p* > 0.20) and *stimulation* (*p* > 0.51) nor a significant *time x stimulation* interaction (*p* > 0.21) at alpha, beta and high gamma frequency.

**Figure 6:**
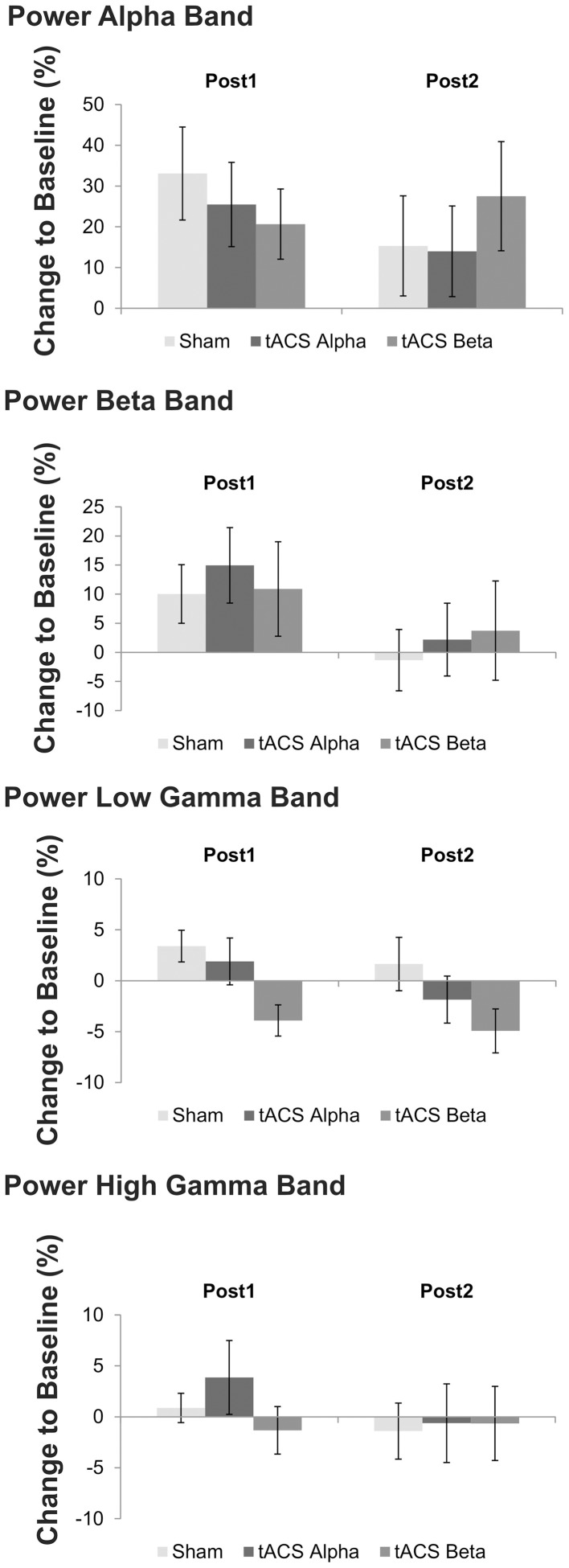
**Mean relative changes of power at the four frequency bands of interest following 10 Hz, 20 Hz and sham tACS**. Error bars indicate standard error of mean. Please note that a trend toward a significant main effect of factor *stimulation* was found on low gamma band power, only. *Post-hoc* analysis revealed that low gamma band power following 20 Hz tACS was significantly reduced as compared to sham stimulation and 10 Hz tACS.

At low gamma frequency a trend toward a significant *stimulation* effect was found [*F*_(2, 22)_ = 3.15, *p* = 0.087]. *Post-hoc t*-tests suggest reduced power at low gamma following 20 Hz tACS as compared to 10 Hz tACS [*t*_(11)_ = 2.26, *p* = 0.04] and sham stimulation [*t*_(11)_ = −3.13, *p* = 0.01]. Figure 6 summarizes mean power changes following tACS at the two post tACS time points. Neither a main effect of factor *time* [*F*_(1, 11)_ = 1.86, *p* = 0.20] nor a significant *time x stimulation* interaction was found to be significant [*F*_(1, 11)_ = 1.23, *p* = 0.30].

During rest the analysis did not reveal a significant main effect (*p* > 0.15) or interaction (*p* > 0.17).

To test whether tACS effects on low gamma CMC were modulated by alpha power, Pearson correlation between both measures was calculated not revealing a significant relation (*R* = −0.039; *p* = 0.82).

## Discussion

The present study aimed at investigating the modulatory offline effects of 10 Hz and 20 Hz tACS on CMC and motor-cortical power during isometric contraction and rest in healthy subjects. Prior to stimulation a significant increase of CMC amplitude during isometric contraction compared to rest was prominent at the beta (13–30 Hz) and low gamma band (30–45 Hz), only. Hence further analysis of tACS effects on CMC was restricted to these frequency bands, while analysis of power was extended to the alpha and high gamma frequency. As a main finding of the present study 10 Hz tACS caused a reduction of CMC amplitude in the low gamma band (30–45 Hz), whereas beta band CMC remained unaffected by stimulation. Since a significant effect of tACS on power was not found at the post-tACS time points (2–8 min and 30–38 min), these results suggest that the observed offline effects of tACS on CMC were unrelated to entrainment of cortical oscillations and occur most likely due to plastic alterations.

### TACS offline effects on corticomuscular coherence (CMC)

Although no effects on local oscillatory activity were discernible, offline effects on CMC were found in terms of a reduction of low gamma band CMC after 10 Hz tACS. Once more, the dissociation between power and CMC supports the assumption that CMC and motor-cortical oscillations represent independent entities (Baker and Baker, 2003; Pollok et al., 2012).

According to Pogosyan et al. (2009) who showed an increase of beta band CMC *during* 20 Hz tACS in a dynamic motor task, we expected to find a frequency-specific modulation of beta band CMC amplitude after 20 Hz stimulation. However, the present data revealed no such effect suggesting that effects of tACS on beta band CMC may be more pronounced online to stimulation when entrainment might be more prominent. Alternatively, this lack of offline effects on beta band CMC might originate from certain sample characteristics. Only subjects who displayed a particularly robust beta band CMC were included in the study. Hence, it might be possible that beta band CMC was more unsusceptible to external modulation via tACS.

Moreover, recent data reveal evidence for the assumption that tACS effects on oscillatory activity might be state dependent (Neuling et al., 2013). Thus, it appears reasonable that application of tACS at rest was not the best solution for eliciting entrainment of cortical oscillations relevant for isometric contraction. We therefore cannot rule out the possibility that tACS *during* isometric contraction might have had stronger offline effects on local oscillations and possibly on CMC after tACS cessation. Nevertheless, the present data suggest that effects on CMC found here are less likely due to entrainment, but may reflect plastic alterations due to tACS.

By means of the 10 Hz tACS offline effect on low gamma band CMC we showed for the first time that such effects are not necessarily limited to stimulation frequency. The interpretation of this finding has to take into account the relevance of gamma band CMC as well as the role of 10 Hz oscillatory activity for motor control. Although the majority of studies describe gamma band CMC in the context of dynamic movement control (Brown and Marsden, 1998; Marsden et al., 2000; Schoffelen et al., 2005; Omlor et al., 2007; Chakarov et al., 2009), the fact that low gamma band CMC increased during isometric contraction compared to rest points to a functional involvement of CMC at this frequency range for static motor control investigated here. This finding is in line with previous studies demonstrating CMC at gamma frequency during isometric contraction (Salenius et al., 1997; Brown, 2000; Gross et al., 2005).

Low gamma band CMC has been associated with binding of visual, proprioceptive, and cognitive (attention) information needed for movement execution (Brown, 2000; Andrykiewicz et al., 2007; Omlor et al., 2007; Patino et al., 2008). In particular, proprioceptive information seems to be essential for the occurrence of gamma band CMC (Patino et al., 2008). Despite not being dynamic in nature, the isometric contraction task applied in the present study requires sensory/proprioceptive reafference and – admittedly to a lesser degree – sustained attention. After all, position of the forearm and contraction strength had to be kept constant over a period of 1 min. Therefore, it seems plausible that even in the present study low gamma band CMC might reflect the integration of sensory/proprioceptive information and attention processes, too.

Increased alpha activity has been associated with cortical idling (Pfurtscheller et al., 1996). Moreover, alpha activity is supposed to contribute to cortical processing via gating through inhibition, blocking the activity in cortical networks irrelevant for the task at hand (Jensen and Mazaheri, 2010). Against this background, one might speculate that 10 Hz tACS might have interfered with the integration of sensory/proprioceptive information and attention-related processes, respectively, by inhibition of information integration finally yielding reduced low gamma band CMC.

An additional line of argument arises from the comparison with findings concerning CMC in PD patients. OFF dopaminergic medication, PD patients exhibit a reduction of CMC during isometric contraction in the beta as well as in the low gamma band (Salenius et al., 2002). Thus, 10 Hz tACS appears to lead to a modulation of functional interaction between cortex and muscle resembling – at least partially – pathological changes in PD. Clinical relevance of increased synchronized activity at 10 Hz for the development of PD symptoms has already been established (Timmermann et al., 2004), indicating that 10 Hz DBS of STN worsens motor symptoms in PD, especially akinesia. Consequently, the present finding of reduced CMC in the low gamma band after 10 Hz tACS might contribute to furthering our understanding of pathological alterations regarding CMC and local oscillatory activity in PD. Irrespective of stimulation location (M1 instead of STN) and although healthy subjects were investigated, we found changes in motor network interaction between cortex and muscle. Based on this finding one might speculate that regardless of stimulation location (cortex or basal ganglia) similar stimulation effects on motor symptoms in PD can be obtained. This assumption might pose a starting point for the future development of noninvasive stimulation alternatives to DBS.

### TACS offline effects on local power

Unexpectedly, we did not find tACS offline effects following 10 and 20 Hz stimulation on local power at alpha or beta frequency. This finding is inconsistent with previous results, demonstrating a frequency-specific enhancement of individual alpha EEG activity after tACS (Zaehle et al., 2010). Additionally, we did not find evidence for the hypothesis that discrete power changes induced by 10 Hz tACS might have yielded the effect on low gamma CMC suggesting that the effects on CMC are indeed unrelated to cortical power alterations. Although speculative at the moment it appears likely that the brief period of movement following tACS might have been detrimental to the retention of potential offline effects on motor-cortical oscillations. On the other hand the present data suggest that following 20 Hz tACS low gamma power was reduced as compared to 10 Hz and sham stimulation. Although this result was marginally significant, it supports the hypothesis that power is modulated by tACS even after stimulation cessation being in line with previous findings (Zaehle et al., 2010; Neuling et al., 2013).

All in all, we would like to stress that although the present data do not reveal frequency entrainment within stimulation frequency after tACS cessation, we would not rule out that such an entrainment might have occurred online concurrently to tACS – not measured here. Interestingly enough, low gamma power was modulated by 20 Hz tACS while CMC at this frequency was affected by 10 Hz stimulation suggesting that effects on CMC cannot be explained by power changes.

## Conclusion

The present study investigated offline effects of 10 and 20 Hz tACS on CMC and local motor-cortical power in healthy subjects. Since 10 Hz tACS was associated with a reduction of low gamma band CMC, the findings suggest cross-frequency interplay between alpha and low gamma band activity modulating functional interaction between motor cortex and muscle. Since alpha power was not affected by 10 Hz tACS the observed offline effect of 10 Hz tACS on low gamma CMC seems to be unrelated to entrainment and may occur due to plastic alterations within the primary sensorimotor cortex.

### Conflict of interest statement

The authors declare that the research was conducted in the absence of any commercial or financial relationships that could be construed as a potential conflict of interest.
